# Does first‐line treatment have prognostic impact for unresectable HCC?—Atezolizumab plus bevacizumab versus lenvatinib

**DOI:** 10.1002/cam4.4854

**Published:** 2022-06-03

**Authors:** Atsushi Hiraoka, Takashi Kumada, Toshifumi Tada, Masashi Hirooka, Kazuya Kariyama, Joji Tani, Masanori Atsukawa, Koichi Takaguchi, Ei Itobayashi, Shinya Fukunishi, Kunihiko Tsuji, Toru Ishikawa, Kazuto Tajiri, Hironori Ochi, Satoshi Yasuda, Hidenori Toyoda, Chikara Ogawa, Takashi Nishimura, Takeshi Hatanaka, Satoru Kakizaki, Noritomo Shimada, Kazuhito Kawata, Atsushi Naganuma, Hisashi Kosaka, Hiroshi Shibata, Tomoko Aoki, Takaaki Tanaka, Hideko Ohama, Kazuhiro Nouso, Asahiro Morishita, Akemi Tsutsui, Takuya Nagano, Norio Itokawa, Tomomi Okubo, Taeang Arai, Michitaka Imai, Yohei Koizumi, Shinichiro Nakamura, Kouji Joko, Hiroko Iijima, Masaki Kaibori, Yoichi Hiasa, Masatoshi Kudo, Real‐life Practice Experts for HCC (RELPEC) Study Group and HCC 48 Group (hepatocellular carcinoma experts from 48 clinics in Japan)

**Affiliations:** ^1^ Gastroenterology Center Ehime Prefectural Central Hospital Matsuyama Japan; ^2^ Department of Nursing Gifu Kyoritsu University Ogaki Japan; ^3^ Department of Internal Medicine Japanese Red Cross Himeji Hospital Himeji Japan; ^4^ Department of Gastroenterology and Metabology Ehime University Graduate School of Medicine Toon Japan; ^5^ Department of Gastroenterology Okayama City Hospital Okayama Japan; ^6^ Department of Gastroenterology and Hepatology Kagawa University Kagawa Japan; ^7^ Division of Gastroenterology and Hepatology, Department of Internal Medicine Nippon Medical School Tokyo Japan; ^8^ Department of Hepatology Kagawa Prefectural Central Hospital Takamatsu Japan; ^9^ Department of Gastroenterology Asahi General Hospital Asahi Japan; ^10^ Department of Gastroenterology Osaka Medical and Phamaceutical University Osaka Japan; ^11^ Center of Gastroenterology Teine Keijinkai Hospital Sapporo Japan; ^12^ Department of Gastroenterology Saiseikai Niigata Hospital Niigata Japan; ^13^ Department of Gastroenterology Toyama University Hospital Toyama Japan; ^14^ Hepato‐Biliary Center Japanese Red Cross Matsuyama Hospital Matsuyama Japan; ^15^ Department of Gastroenterology and Hepatology Ogaki Municipal Hospital Ogaki Japan; ^16^ Department of Gastroenterology Japanese Red Cross Takamatsu Hospital Takamatsu Japan; ^17^ Department of Gastroenterology and Hepatology Hyogo College of Medicine Hyogo Japan; ^18^ Department of Gastroenterology Gunma Saiseikai Maebashi Hospital Gunma Japan; ^19^ Department of Clinical Research National Hospital Organization Takasaki General Medical Center Takasaki Japan; ^20^ Division of Gastroenterology and Hepatology Otakanomori Hospital Kashiwa Japan; ^21^ Hepatology Division, Department of Internal Medicine II Hamamatsu University School of Medicine Hamamatsu Japan; ^22^ Department of Gastroenterology National Hospital Organization Takasaki General Medical Center Takasaki Japan; ^23^ Department of Surgery Kansai Medical University Hirakata Japan; ^24^ Department of Gastroenterology Tokushima Prefectural Central Hospital Tokushima Japan; ^25^ Department of Gastroenterology and Hepatology Kindai University Faculty of Medicine Osaka Japan

**Keywords:** atezolizumab plus bevacizumab, hepatocellular carcinoma, lenvatinib, prognosis

## Abstract

**Background/Aim:**

A comparison of therapeutic efficacy between atezolizumab plus bevacizumab (Atez/Bev) and lenvatinib treatment given as first‐line therapy for unresectable hepatocellular carcinoma (u‐HCC) in regard to progression‐free survival (PFS) overall survival (OS) has not been reported. We aimed to elucidate which of those given as initial treatment for u‐HCC has greater prognostic impact on PFS and OS of affected patients, retrospectively.

**Materials/Methods:**

From 2020 to January 2022, 251 u‐HCC (Child–Pugh A, ECOG PS 0/1, BCLC‐B/C) treated were enrolled (Atez/Bev‐group, *n* = 194; lenvatinib‐group, *n* = 57). PFS and OS were analyzed following adjustment based on inverse probability weighting (IPW).

**Results:**

There was a greater number of patients with macro‐vascular invasion in Atez/Bev‐group (22.7% vs. 8.8%, *p* = 0.022). In lenvatinib‐group, the frequencies of appetite loss (38.6% vs. 19.6%, *p* = 0.002), hypothyroidism (21.1% vs. 6.7%, *p* = 0.004), hand foot skin reaction (19.3% vs. 1.0%, *p* < 0.001), and diarrhea (10.5% vs. 4.6%, *p* = 0.012) were greater, while that of general fatigue was lower (22.8% vs. 26.3%, *p* = 0.008). Comparisons of therapeutic best response using modified response evaluation criteria in solid tumors (mRECIST) did not show significant differences between the present groups (Atez/Bev vs. lenvatinib: CR/PR/SD/PD = 6.1%/39.1%/39.1%/15.6% vs. 0%/48.0%/38.0%/14.0%, *p* = 0.285). In patients of discontinuation of treatments, 48.2% switched to lenvatinib, 10.6% continued beyond PD, 8.2% received another systemic treatment, 5.9% underwent transcatheter arterial chemoembolization (TACE), 3.5% received hepatic arterial infusion chemotherapy (HAIC), and 1.2% underwent surgical resection in Atez/Bev‐group, while 42.2% switched to Atez/Bev, 4.4% continued beyond PD, 4.4% received another systemic treatment, 2.2% nivolumab, 6.7% received TACE, and 2.2% received HAIC in lenvatinib‐group. Following adjustment with inverse probability weighting (IPW), Atez/Bev‐group showed better PFS (0.5−/1−/1.5‐years: 56.6%/31.6%/non‐estimable vs. 48.6%/20.4%/11.2%, *p* < 0.0001) and OS rates (0.5−/1−/1.5‐years: 89.6%/67.2%/58.1% vs. 77.8%/66.2%/52.7%, *p* = 0.002).

**Conclusion:**

The present study showed that u‐HCC patients who received Atez/Bev as a first‐line treatment may have a better prognosis than those who received lenvatinib.

## Graphical abstract

This is a first report mentions that a comparison of therapeutic efficacy between atezolizumab plus bevacizumab (Atez/Bev) and lenvatinib treatment given as first‐line therapy for unresectable hepatocellular carcinoma (u‐HCC) in regard to overall survival (OS). Although tumor responses did not show significant differences between both groups, Atez/Bev‐group showed better OS rates following adjustment with IPW (0.5−/1−/1.5‐years: 89.6%/67.2%/58.1% vs. 77.8%/66.2%/52.7%, *p* = 0.002). Atez/Bev‐group showed better OS as a first‐line treatment as compared to lenvatinib‐group.

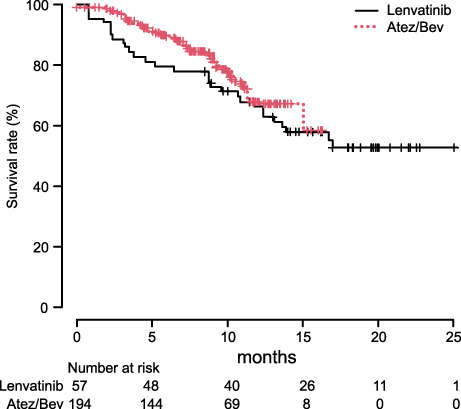

## 1 INTRODUCTION

Hepatocellular carcinoma (HCC) is one of the major malignancies and reported to be fifth most common worldwide.^1^ Additionally, recurrence following curative treatment (e.g., surgical resection, radiofrequency ablation [RFA]) is often seen, with the tumor finally showing an unresectable state in many of those cases, even when hepatic reserve function is maintained.

Following development of the tyrosine kinase inhibitor (TKI) sorafenib^2^ as the first systemic treatment medication, a total of three regimens are available at the time of writing for first‐line treatment. Lenvatinib^3^ was the second first‐line therapy drug to receive approval, based on results showing its non‐inferiority therapeutic efficacy as compared with sorafenib, and that has since been used for initial systemic treatment because of the powerful therapeutic response noted in affected patients. Thereafter, atezolizumab plus bevacizumab (Atez/Bev) treatment^4^ received approval in September 2020 as a first‐line treatment regimen based on its superior therapeutic effects for progression‐free survival (PFS) by response evaluation criteria in solid tumors ver. 1.1 (RECIST)^5^ and overall survival (OS) for unresectable HCC (u‐HCC) as compared with sorafenib. Recently, Kudo et al.^6^ and Tada et al.^7^ have reported that therapeutic usefulness of lenvatinib as an initial therapy other than transcatheter arterial chemoembolization (TACE) for intermediate stage u‐HCC, respectively [Barcelona Clinic Liver Cancer stage B (BCLC‐B)^8^]. However, to the best of our knowledge, although there has only one report of Atez/Bev for BCLC‐B u‐HCC has been reported,^9^ there have been no studies that have been conducted to compare therapeutic efficacy related to OS between Atez/Bev and lenvatinib when given as first‐line treatment for u‐HCC. The present retrospective study aimed to elucidate which of those given as initial treatment for u‐HCC has greater prognostic impact on OS of affected patients.

## 2 MATERIALS/METHODS

**FIGURE 1:**
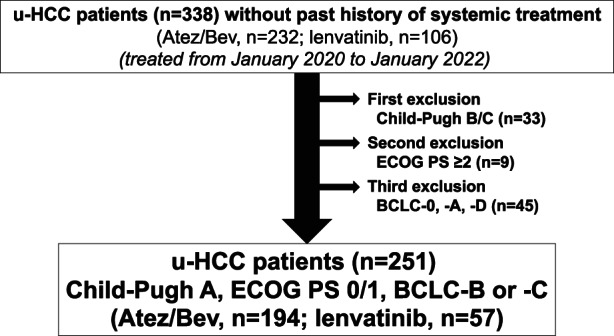
Patients' enrollment flow.

The present study was set to elucidate differences of therapeutic results between u‐HCC patients with Atez/Bev and lenvatinib (before Atez/Bev approval) as an initial treatments for all u‐HCC patients, and examined the records of 338 u‐HCC patients (Atez/Bev: lenvatinib = 232:106) treated between January 2020 and January 2022. All patients treated with lenvatinib as a first‐line therapy were started treatment prior to Atez/Bev approval (September 2020). The above enrollment period was set because the median PFS of lenvatinib treatment in u‐HCC patients with Child–Pugh A was 7.8 months (95% Confidence interval [CI]: 7.0–8.0)^10^ and it was thought that it would theoretically be more likely that Atez/Bev would be used as a post progression therapy of lenvatinib and vice versa, and that crossover with Atez/Bev as a post progression therapy of lenvatinib would be common in the clinical setting. Following exclusion of 87 classified as Child–Pugh B or C (*n* = 33), an Eastern Cooperative Oncology Group Performance Status (ECOG PS) ≥2 (*n* = 9), or with BCLC‐0, −A, or ‐D (*n* = 45), 251 patients without a past history of systemic treatment, and treated with Atez/Bev (*n* = 194) or lenvatinib (*n* = 57) at the standard dose at our affiliated hospitals were enrolled (Figure 1).

Prior to starting therapy, written informed consent was obtained from each patient. Intravenous Atez/Bev treatment consisted of atezolizumab (1200 mg) plus bevacizumab (15 mg/kg) of body weight (BW) and was given every 3 weeks,^4^ based on the treatment guidelines for Atez/Bev provided by the manufacturer. As for lenvatinib treatment, that was orally administered (BW <60 kg: 8 mg/day, or BW ≥60 kg: 12 mg/day). When any unacceptable or serious adverse event (AE) or clinical tumor progression was observed, lenvatinib treatment was discontinued. When a patient developed severe AE (≥grade 3), or if any unacceptable grade 2 drug‐related AE occurred, the drug dose was reduced or treatment interrupted according to the administration guidelines for Atez/Bev and lenvatinib. When a drug‐related AE was noted, dose reduction or temporary interruption was maintained until the symptom was resolved to grade 1 or 2, according to the guidelines provided by the manufacturer. Treatment was discontinued following observation of any unacceptable or serious AE, or clinical tumor progression. Each patient was examined using upper gastrointestinal endoscopy for surveillance to detect gastroesophageal varices within 6 months of introduction of Atez/Bev. When bleeding was detected or in cases with high risk, the patient was treated according to local clinical practice.

### 2.1 Underlying liver disease

HCC due to hepatitis B virus (HBV) was determined when the HBV surface antigen was positive, whereas positive anti‐hepatitis C virus (HCV) findings were considered to indicate that HCC was due to HCV. Underlying liver disease was judged as related to alcohol, when patients had a history of alcohol abuse (≥60 g/day).^11^, ^12^ Patients had a known history of autoimmune disease, they were not treated with Atez/Bev.

### 2.2 Diagnosis of HCC

Diagnosis of HCC was based on an increasing course of alpha‐fetoprotein (AFP), as well as dynamic‐computed tomography (CT),^13^ magnetic resonance imaging (MRI),^5^, ^14^ and/or pathological findings obtained during the clinical course. BCLC^8^ was used for tumor progression status.

### 2.3 Response evaluation

In patients treated with Atez/Bev, the modified RECIST (mRECIST), ver. 1.1,^5^ was used for evaluation of therapeutic response [complete response (CR), partial response (PR), stable disease (SD), progressive disease (PD)].

The initial assessment of therapeutic efficacy was performed using dynamic‐CT at approximately 6 weeks after Atez/Bev introduction, whenever possible, then additional dynamic‐CT examinations were performed as needed depending on patient condition, even before 6 weeks in some cases. After the initial 6 weeks, dynamic‐CT examinations were performed again every 6 weeks and then every 9 to 12 weeks after the first 6 months. In the lenvatinib group, the initial assessment was performed at 4 weeks after introduction of the drug and then examinations were performed at intervals of 8–12 weeks thereafter.

### 2.4 Assessment for liver function

Child–Pugh classification,^15^ albumin‐bilirubin (ALBI) grade,^16^, ^17^ and modified ALBI (mALBI) grade,^18^ for which ALBI grade 2 was divided into two sub‐grades (mALBI 2a and 2b) using an ALBI score of −2.27 as the cutoff value, were used.

### 2.5 Assessment of adverse events during Atez/Bev and lenvatinib treatments

For assessment of AEs, the National Cancer Institute Common Terminology Criteria for Adverse Events, ver. 5.0,^19^ was used. At the time of Atez/Bev discontinuation, introduction of the next treatment was determined by the attending physician.

The present study was conducted after receiving official approval, as a retrospective analysis of database records based on the Guidelines for Clinical Research issued by the Ministry of Health and Welfare of Japan. After receiving written informed consent from each of the enrolled patients, all procedures were done in accordance with the declaration of Helsinki.

### 2.6 Statistical analysis

Continuous variables are expressed as median values (interquartile range [IQR]). For statistical analyses, Student's *t*‐test, Welch's *t*‐test, a Mann–Whitney *U* test, the Kaplan‐Meier method, and a log‐rank test were used. Atez/Bev and lenvatinib group probabilities (propensity) were calculated using logistic regression analysis with a set of covariates deemed likely to have effects on OS, including positive for macro‐vascular invasion (MVI) and alanine aminotransferase (ALT), clinical factors with a low *p* value (*p* < 0.1) (Table 1). Inverse probability weighting (IPW) was defined as 1/(propensity score) for the Atez/Bev group and 1/(1‐propensity score) for the lenvatinib group. Differences regarding OS and PFS were tested using an IPW‐adjusted log‐rank test.^20^, ^21^ *p* values <0.05 were considered to indicate statistical significance. Easy‐R (EZR), ver. 1.53 (Saitama Medical Center, Jichi Medical University),^22^ a graphical user interface for R (The R Foundation for Statistical Computing), was used to perform all of the statistical analyses.

**TABLE 1:** Characteristics of Atez/Bev and lenvatinib groups

	Lenvatinib (*n* = 57)	Atez/Bev (*n* = 194)	*p* value
Age, years^a^	73 (69–79)	74 (68–79)	0.870
Gender, male: female	41:16	148:46	0.490
ECOG PS, 0:1	47:10	167:27	0.525
Body mass index, kg/m^2^ ^a^	23.7 (21.8–26.6)	23.9 (21.6–26.1)	0.211
Etiology, HCV:HBV:alcohol:others	24:312:18	70:32:33:59	0.154
AST, U/L^a^	33 (21–45)	38 (26–57)	0.424
ALT, U/L^a^	33 (21–45)	26 (18–40)	0.081
Platelets, 10^4^/μl^a^	15.1 (110.6–21.0)	13.8 (10.5–20.4)	0.802
T‐bilirubin, mg/dl^a^	0.70 (0.60–0.97)	0.80 (0.60–1.00)	0.211
Albumin, g/dl^a^	3.8 (3.6–4.1)	3.8 (3.5–4.2)	0.789
Prothrombin time, %^a^	89.5 (81.5–99.3)	88.4 (81.3–97.4)	0.604
eGFR, ml/min/1.73 m^2^ ^a^	65.7 (44.3–82.0)	66.9 (52.6–78.2)	0.911
ALBI score at baseline^a^	−2.57 (−2.28 to −2.79)	−2.52 (−2.23 to −2.76)	0.661
mALBI 1:2a:2b	27:16:14	84:53:57	0.779
Child–Pugh score 5:6	39:18	133:61	1.0
Maximum intrahepatic tumor size, ≥5 cm (%)	20 (35.1%)	71 (36.6%)	0.877
No. of intrahepatic tumors, none:single:multiple	3:8:46	11:30:153	1.0
Positive for MVI (%)	5 (8.8%)	44 (22.7%)	0.022
Positive for EHM (%)	15 (26.3%)	71 (36.6%)	0.158
BCLC B:C	34:23	93:101	0.134
AFP (≥400 ng/ml) (%)	15 (26.3%)	44 (22.7%)	0.596
No past history of treatment for HCC (%)	8 (14.0%)	45 (23.2%)	0.195
Died (%)	23 (40.4%)	35 (18.0%)	0.001
Observation period, months^a^	14.4 (9.3–19.0)	8.0 (4.9–11.2)	<0.001
IPW^a^	3.91 (3.43–4.26)	1.31 (1.18–1.38)	<0.001

Abbreviations: AFP, alpha‐fetoprotein; ALBI score, albumin‐bilirubin score; ALT, alanine aminotransferase; AST, aspartate transaminase; Atez/Bev, atezolizumab plus bevacizumab treatment; BCLC, Barcelona Clinic Liver Cancer stage; ECOG PS, Eastern Cooperative Oncology Group Performance Status; EHM, extrahepatic metastasis; HBV, hepatitis B virus; HCV, hepatitis C virus; IPW, inverse probability weighting; mALBI grade, modified ALBI grade; MVI, macro‐vascular invasion.

^a^ Median. Values in parentheses show interquartile range, unless otherwise indicated.

## 3 RESULTS

In comparisons of the Atez/Bev and lenvatinib groups, no significant differences were observed in regard to clinical features, except for frequency of MVI [22.7% (44/194) vs. 8.8% (5/57), *p* = 0.022], and observation period [median 8.0 months (IQR: 4.9–11.2) vs. 14.4 months (IQR: 9.3–19.0), *p* < 0.001] (Table 1). AEs that occurred greater than 10% of the patients in at least one of the groups (any grades) are shown in Table 2. In the lenvatinib group, the frequencies of appetite loss (38.6% vs. 19.6%, *p* = 0.002), hypothyroidism (21.1% vs. 6.7%, *p* = 0.004), hand foot skin reaction (19.3% vs. 1.0%, *p* < 0.001), and diarrhea (10.5% vs. 4.6%, *p* = 0.012) were greater, while that of general fatigue was lower (22.8% vs. 26.3%, *p* = 0.008) as compared to the Atez/Bev group.

**TABLE 2:** Adverse events in greater than 10% of patients in at least one group (all grades)

	Lenvatinib group (*n* = 57)	Atez/Bev group (*n* = 194)	*p* value
Appetite loss (grade 0:1:2:3)	35:5:13:4	156:20:13:5	0.002
General fatigue (grade 0:1:2:3)	44:2:11:0	143:31:16:4	0.008
Protein urine (grade 0:1:2:3)	43:5:5:4	148:13:14:19	0.824
Hypothyroidism (grade 0:1:2:3)	45:3:9:0	181:4:7:2	0.004
Hypertension (grade 0:1:2:3)	48:2:6:1	160:10:15:9	0.746
HFSR (grade 0:1:2:3)	46:6:4:1	192:1:1:0	<0.001
Hepatic examination abnormality (grade 0:1:2:3:4)	56:0:1:0:0	172:12:7:2:1	0.261
Diarrhea (grade 0:1:2:3)	51:1:5:0	185:7:2:0	0.012
Platelet count decline (grade 0:1:2:3)	50:2:4:1	185:3:5:1	0.120
Edema/ascites (grade 0:1:2:3)	55:1:0:1	173:7:12:2	0.144
Rash (grade 0:1:2:3)	55:2:0:0	174:10:8:2	0.446

Abbreviations: Atez/Bev, atezolizumab plus bevacizumab treatment; HFSR, hand‐foot skin reaction.

Comparisons of therapeutic best response by mRECIST found no significant differences between the groups [Atez/Bev vs. lenvatinib: CR/PR/SD/PD, 11:70:70:28 (objective‐response rate (ORR)/disease‐control rate (DCR) = 45.2%/84.4%) vs. 0:24:19:7 (ORR/DCR = 48.0%/86.0%), *p* = 0.285]. When therapeutic efficacies were evaluated by RECIST ver. 1.1,^23^ no significant differences were observed between the groups [Atez/Bev vs. lenvatinib: CR/PR/SD/PD by RECIST, 4.0%/25.6%/48.2%/16.1% (ORR/DCR = 29.6%/77.9%) vs. 0%/19.6%/51.8%/16.1% (ORR/DCR = 19.6%/83.9%), *p* = 0.270].

**FIGURE 2:**
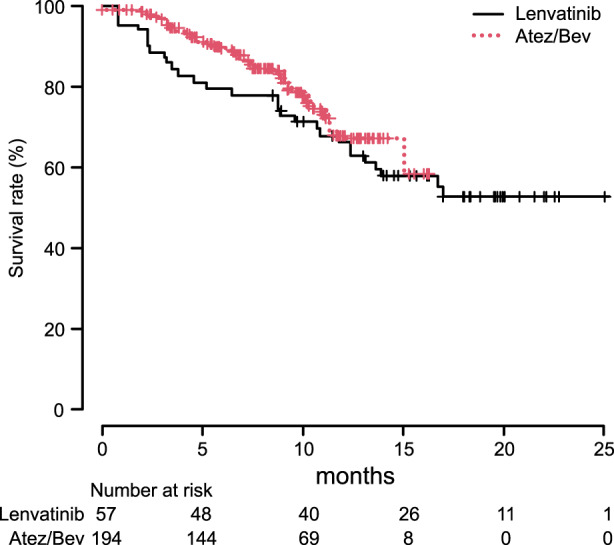
Overall survival with adjustment using inverse probability weighting. The Atez/Bev group showed better overall survival (OS) as compared to the lenvatinib group after 0.5, 1, and 1.5 years (OS rate: 89.6%/67.2%/58.1% vs. 77.8%/66.2%/52.7%, *p* = 0.002).

**FIGURE 3:**
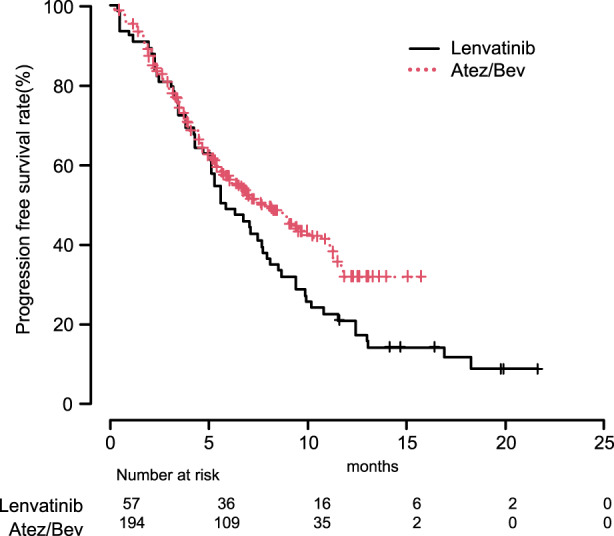
Progression‐free survival by modified RECIST with adjustment using inverse probability weighting. The Atez/Bev group showed better progression‐free survival (PFS) by modified RECIST as compared to the lenvatinib group after 0.5, 1, and 1.5 years (PFS rate: 56.6%/31.6%/non‐estimable (NE) vs. 48.6%/20.4%/11.2%, *p* < 0.0001).

When OS and PFS by mRECIST were analyzed without using IPW, no significant differences were observed between the Atez/Bev and lenvatinib groups [median OS: non‐estimable (NE) ((95%CI: 15.0‐ not applicable [NA]) vs. NE (95%CI: 13.6‐NA), *p* = 0.435); median PFS: 8.0 months (95%CI: 6.2–10.3) vs. 6.8 months (95%CI: 4.8–8.1), *p* = 0.109]. On the other hand, when OS and PFS by mRECIST were analyzed using IPW, the Atez/Bev group showed a better OS rate after 0.5, 1, and 1.5 years (89.6%/67.2%/58.1% vs. 77.8%/66.2%/52.7%, *p* = 0.002) (Figure 2), as well as better PFS rate after 0.5, 1, and 1.5 years (56.6%/31.6%/NE vs. 48.6%/20.4%/11.2%, *p* < 0.0001) (Figure 3).

At the time of analysis, 56.2% (109/194) of the Atez/Bev group and 21.1% (12/57) in the lenvatinib group were receiving ongoing treatment. Major reasons for discontinuation of the initial systemic treatment in the Atez/Bev group were PD in 66 (77.6%), and AEs in 24 (28.2%), and those were PD in 36 (80.0%), and AEs in four (8.8%) in the lenvatinib group (there were duplicated cases). Post progression treatments were analyzed in patients for whom the initial systemic therapy was abandoned due to treatment failure. In 85 patients of discontinuation of the Atez/Bev group, 41 (48.2%) switched to lenvatinib, nine (10.6%) continued Atez/Bev beyond PD, seven (8.2%) received another TKI/molecular targeting agent (MTA) (sorafenib/ramucirumab), five (5.9%) underwent TACE, three (3.5%) received hepatic arterial infusion chemotherapy (HAIC), one (1.2%) underwent surgical resection, one (1.2%) radio‐therapy (RT), 17 (20.0%) received best supportive care (BSC), and one (1.2%) was unknown. In Atez/Bev group, migration rate to post progression treatment other than BSC, RT and unknown was 77.6%. In 45 patients of discontinuation of the lenvatinib group, 19 (42.2%) switched to Atez/Bev, two (4.4%) continued lenvatinib beyond PD, two (4.4%) received another TKI/MTA (sorafenib/ramucirumab), one (2.2%) joined a clinical trial (nivolumab), three (6.7%) received TACE, one (2.2%) received HAIC, 11 (24.4%) received BSC, one (2.2%) underwent mass reduction radiofrequency ablation (RFA), and five (11.1%) were unknown. In lenvatinib group, migration rate to post progression treatment other than BSC, mass reduction RFA and unknown was 62.2%.

## 4 DISCUSSION

This is a first report, which compares therapeutic efficacies between Atez/Bev and lenvatinib as an initial therapy for u‐HCC. Patients treated with Atez/Bev showed superior OS and PFS values as compared to those treated with lenvatinib, after adjusting with IPW. The present results were thought to be based not only on better PFS, but also because of patients with existing CR or conversion treatment in the Atez/Bev group. Although, the discontinuation rate due to AEs of Atez/Bev was higher in the present cohort (21.2%) than that of the lenvatinib group (8.5%), it has been reported that significant deterioration of ALBI score is common during the initial 4 weeks after introducing lenvatinib treatment,^24^ while a lower negative influence of Atez/Bev on hepatic function^9^, ^25^ might have contributed to the present results. It is no doubt that introduction of not only a TKI or MTA^26^, ^27^, ^28^, ^29^, ^30^ but also Atez/Bev in subjects with better hepatic function condition (mALBI grade 1 or 2a) is considered to be a minimum requirement to improve prognosis.

Following Atez/Bev failure, it is considered that use of a TKI or MTA (i.e., regorafenib,^31^ ramucirumab,^32^ and cabozantinib,^33^, ^34^ each available in Japan in January 2022, in addition to sorafenib and lenvatinib) with sequential systemic treatments will have an increasingly vital role to improve prognosis. In cases that received TKI treatment, Child–Pugh class A patients have been reported to have a better post progression survival (PPS) as compared to those classified as Child–Pugh class B patients (54.4 ± 17.6% vs. 32.0 ± 11.6%, *p* = 0.015).^35^ Moreover, with TKI and MTA sequential treatment, the total TKI/MTA therapy period in patients treated after 2017 was found to have a good correlation with OS in our previous study (*r* = 0.946, 95% CI: 0.918–0.965, *p* < 0.001),^27^ while Kobayashi et al. reported that the correlation between OS and duration of TKI/MTA has improved with the increase in therapeutic modalities in recent years (2009–2012 vs. 2013–2016 vs. 2017–2019: *R* = 0.395 vs. 0.505 vs. 0.667).^36^

To increase the opportunity for prolonging PPS by sequential therapy, introduction of Atez/Bev for patients with better hepatic function should be kept in mind as much as possible. In the present cohort, migration to post progression treatment (including continuing beyond PD and interventional radiology) was noted in 77.6% of the Atez/Bev group, similar to the ranges (74.2% to 88.2%) noted in previous reports presented by Hayakawa et al.^37^ and Yoo et al.^38^ This is the same as findings showing that post progression treatment with TKI/MTA and continuing treatment including such treatments in a sequential manner is very important to prolong the prognosis of u‐HCC patients.

In recent reports, a better therapeutic efficacies were obtained by lenvatinib treatment as an initial therapy for BCLC‐B u‐HCC (beyond up to seven criteria) as compared to TACE.^6^, ^7^ On the other hand, although a good therapeutic results of Atez/Bev for patients with such condition were observed,^9^ there have been no other reports concerning with comparison of therapeutic efficacies for u‐HCC between Atez/Bev and lenvatinib. The present results of this retrospective study suggested the superiority of Atez/Bev to lenvatinib as an initial treatment for both BCLC‐B and ‐C u‐HCC patients. Although the rate of migration to post progression treatment of the lenvatinib group (including TACE and continuing beyond PD) was 62.2% in the present study, in past investigations of lenvatinib treatment, low rates of eligibility for post progression therapy (43.8%),^39^ and of migration from lenvatinib to second‐line treatment (41.7%)^40^ were reported, with lack of an established post progression treatment also noted as an important clinical issue. In spite of such clinical factors, it has been reported that later‐line use of lenvatinib showed a similar therapeutic efficacy to that of the initial use.^10^ In addition, Aoki et al.^41^ reported that the median OS after introducing lenvatinib as a post progression treatment of ICI therapy was 15.8 months (95%CI: 8.49–23.17), while Yoo et al.^38^ noted that after Atez/Bev failure was 16.6 months (95%CI: 3.6–29.6). In the present study, 56.2% of the Atez/Bev group showed a treatment ongoing status at the time of writing, while approximately 40%–50% of the patients with treatment failure (PD) crossed over to receive the other treatment. Although further investigation of OS is needed because the present observation period was short, Atez/Bev showed superiority to LEN in terms of PFS.

There are some limitations. First is its retrospective nature. Second, the timing for introduction of lenvatinib was prior to development of Atez/Bev. Third, the observation period in the Atez/Bev group was short. To obtain clear conclusion, longer observation period will be needed. Additional investigations including a randomized control study with a larger number of patients are needed in the near future.

The present study showed that u‐HCC patients treated with Atez/Bev as a first‐line therapy may have a better prognosis than those who received lenvatinib. In the present immune therapy era, the most effective sequential order of TKI/MTA following Atez/Bev failure should be elucidated. Furthermore, clear therapeutic strategies of systemic treatments must be established to improve prognosis for affected patients.

## AUTHOR CONTRIBUTIONS

AH and TK conceived the study, and participated in its design and coordination. AH, TTad, MH, KKar, MA, JT, KTa, EI, KTs, SF, TI, KTaj, HO, CO, SY, HT, TNi, TA, SK, TH, NS, KKaw, MKa, AN, TTan, HO, KN, HS, AM, AT, TNa, NI, TO, TA, YK, MI, SN, HI, YH, KJ, HK, and MKu performed data curation. AH performed statistical analyses and interpretation. AH and TK drafted the text. All authors have read and approved the final version of the manuscript.

## FUNDING INFORMATION

None.

## CONFLICT OF INTEREST

Atsushi Hiraoka, MD, PhD – lecture fees: Chugai, Eli Lilly, Bayer. Takashi Kumada, MD, PhD – lecture fees; Eisai. Masatoshi Kudo, MD, PhD – advisory role: Eiasi, Ono, MSD, Bristol‐Myers Squibb, Roche; lecture fees: Bayer, Eli Lilly, Eisai, MSD, Bristol‐Myers Squibb, EA Pharma; research funding: Gilead Sciences, Taiho, Sumitomo Dainippon Pharma, Otsuka, Takeda, EA Pharma, Abbvie, Eisai. Toshifumi Tada, MD, PhD – lecture fees; Eisai, Abbvie. Takeshi Hatanaka, MD, PhD – lecture fee; Eisai.

None of the other authors have potential conflict of interest to declare.

## ETHICS STATEMENT

The entire study protocol was approved by the Institutional Ethics Committee of Ehime Prefectural Central Hospital (No. 30–66). The present study was conducted after receiving official approval as a retrospective analysis of database records based on the Guidelines for Clinical Research issued by the Ministry of Health and Welfare of Japan. All procedures were done in accordance with the declaration of Helsinki. The data were made anonymous before analysis to protect patient privacy. Written informed consent was obtained from all patients before treatment and ethical approval for use of an opt‐out methodology was received for this study based on the low level of risk to the participants.

## Data Availability

Due to the nature of this research, the participants in this study could not be contacted regarding permission for the findings to be shared publicly, thus no such permission was asked for or obtained. The datasets generated and/or analyzed for the current study are not publicly available due to the nature of the research, as noted above.
